# Molluscs production associated to lunar-tide cycle: a case study in Paraíba State under ethnoecology viewpoint

**DOI:** 10.1186/1746-4269-2-28

**Published:** 2006-06-19

**Authors:** Alberto K Nishida, Nivaldo Nordi, Rômulo RN Alves

**Affiliations:** 1 Departamento de Sistemática e Ecologia, Universidade Federal da Paraíba, 58051-900 João Pessoa, PB, Brasil; 2 Departamento de Hidrobiologia, Universidade Federal de São Carlos, Rod. Washington Luiz, Km 235, 13565-905 São Carlos, SP, Brasil; 3 Departamento de Biologia, Universidade Estadual da Paraíba, Av. das Baraúnas, 351/Campus Universitário, Bodocongó, 58109-753, Campina Grande, PB, Brasil; 4 Programa de Pós-Graduação em Ciências Biológicas (Zoologia), Departamento de Sistemática e Ecologia, Universidade Federal da Paraíba, 58059-970 João Pessoa, PB, Brasil

## Abstract

Molluscs have been for a long time a very important food resource for humans. Therefore, oysters, clams, and mussels are highly required at seafood markets. Like any commercial food, it is necessary that molluscs present good quality standards, concerning some criteria such as amount of meat and appearance. In bivalves, condition index or fattening index is considered a satisfactory method for estimating the amount of meat related to the shell cavity. Molluscs gatherers of Paraíba State coast, northeastern Brazil, state that molluscan meat production increases during spring tide (designated by them as *maré de lançamento*) in opposition to the meat decrease which happens during neap tide (*maré de quebramento*) (they are designated technically in Portuguese as *maré de sizígia *and *maré de quadratura*, respectively). Weperformed a survey on the production of *unha-de-velho *or 'oldman'snail' (*Tagelus plebeius*) caught by molluscs gatherers in the estuary of River Paraíba do Norte, by observing locally their work, applying questionnaires, searching for a possible scientific relation of that molluscs condition to the gatherers empirical statement. Thus, we estimatedthe molluscs condition index through the method of solids percentage determination. We studied their work and the molluscs condition index during a full lunar-tide cycle. Determinations were carried out between 2nd September and 20th October, 1998, through 20 catches performed to obtain condition index from 400 bivalves. We observed that several biotic and abiotic ecological factors, namely reproduction cycle, biochemical components variations, animal size, and even parasitism, may affect the animal condition index. Despite this aspect, our present results confirmed a high overlapping (80%) of the condition index curve with lunar-tide cycle, in agreement with the gatherers statement. Although we recognize the need for formulating and testing other hypotheses, we consider *a priori *that the gatherers empirical assertion *a unha tá gorda de acordocom a maré *('the "oldman's nail" is fat according to the tide', roughly translating) is justified by the observations here performed when the condition index increased during spring tide and decreased during neap tide.

## Introduction

Mangrove estuarine areas often support an abundance of mollusc species thatare largely sessile in nature and constitute an important in-situ fishery [1-4]. Edible species of oysters, mussels, cockles, and gastropods are collected extensively for local consumption, usually by the families of local fishermen [5]. The ethnoecological knowledge of molluscs has been reported by Vannuci [6], who stated that people from Milingimbi, Australia, are able to identify about eighty bivalve species and classify "naturally" the local fauna in a way that reflects the habits, availability, associations, and ecophysiology of those species, especially the edible ones and those with other uses.

Prehistoric deposits of shells, skeletons and refuse on the Brazilian coastnear estuaries, known as 'sambaquis' [7], demonstrate the early use of molluscs by human populations. In Brazil, mollusc gatherers are economically marginal human communities; nevertheless they have a deep knowledge of the bioecology of these animals and on environmental factors affecting them. This sort of knowledge may support management plans, especially of highly exploited resources [8].

Human communities relying directly on their natural resources for subsistence, usually have a detailed knowledge of their environments [9-11]. The economic, social, and cultural activities of those people depend uponthe local biota and the implicit environmental cycles. The daily observations of fishermen on the resources and fishing environments result in an accumulation of knowledge that will support ecological studies [12,13].

Oysters, clams, and mussels are highly required by food markets. As molluscs have been for a long time an important food resource for humans, like anycommercial product they must have good quality standards concerning some criteria such as amount of meat and appearance. In bivalves, condition index or fattening index, or simply condition, is one of most satisfactory evaluation methods for estimating the amount of meat related to the shell cavity.

Molluscs gatherers of Paraíba State coast, whose catching procedures and traditions are aimed at in the present study, state that molluscan production increases during spring tides (*marés de lançamento*, as they designate them in Portuguese, or technically as *marés de sizígia*), because those animals are fatter than during neap tides (*marés de quebramento *also in Portuguese or technically, *marés de quadratura*). In the present work we estimated the production of *unha-de-velho *('oldman's nail') (*Tagelus plebeius*) obtained by molluscs gatherers, aiming to confirm scientificallytheir empirical observation by evaluating their catching procedures and applying questionnaires.

## Methods

### The study area

**Figure 1:**
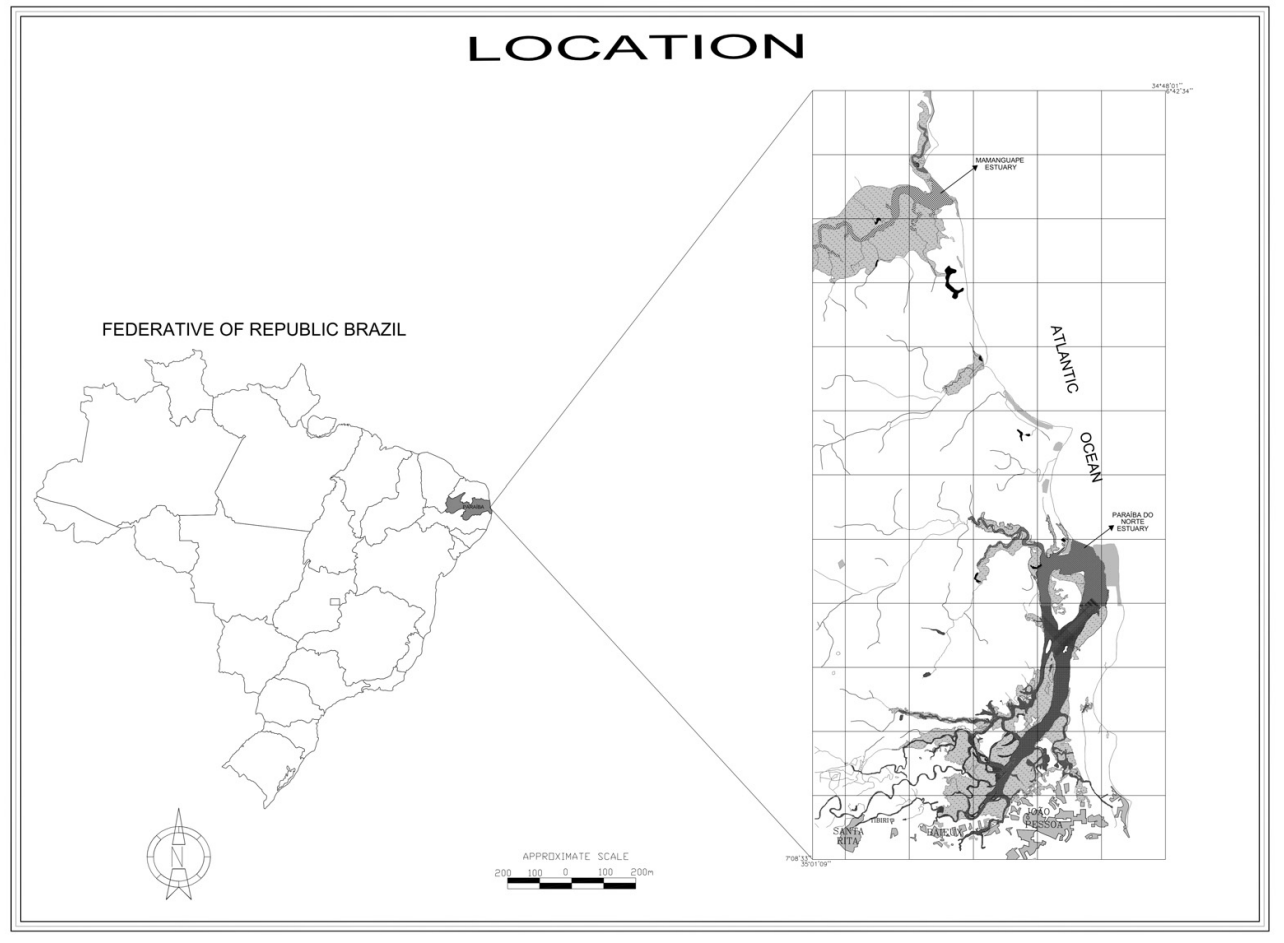
Map of Brazil showing in the inset the coast of Paraíba state with the study area.

The present work was carried out in the estuary of River Paraíba do Norte. The basin of the most important river of the State of Paraíba extends for *ca*. 380 km, crossing 37 municipalities. It is divided in Alto (High), Médio (Median), and Baixo (Low) Paraíba [14]. The Baixo Paraíba is located between lat 60°57' and 70°08' S and between long 34°50' and 34°55' W. It drains areas in the municipalities of João Pessoa (the capital of the State of Paraíba), Bayeux, Santa Rita, and Cabedelo (Fig. 1).

In the last ten years the exploitation of resources from the natural estuary-mangrove system has been intensified mainly due to the increasing population in the periphery of João Pessoa. A large part of the population living on the left bank of the river exploits those resources for food and income. On the right bank, where urbanization has been intensified, there is a reduced number of people which depends on the estuarine resources [15].

### Procedures

The field research was carried by visiting the human community described above. Before starting the field work, it was held a meeting with mollusc gatherers to inform them about our goals and methods of study, and to propose their participation in our investigations. The empirical knowledge of mollusc gatherers regarding bioecology of *Tagelus plebeius *were obtained by applying semi-structured questionnaires, complimented by semi-directive interviews and informal conversations [8]. Twelve mollusc gatherers were interviewed, making up a stratified sampling. Only mollusc gatherers living exclusively from such activity for at least 20 years were interviewed. All mollusc gatherers state that molluscan production increases during spring tides (*marés de lançamento*, as they designate them in Portuguese, or technically as *marés de sizígia*), because those animals are fatter than during neap tides (*marés de quebramento *also in Portuguese or technically, *marés de quadratura*).

Among many classic methods reported by Hickman and Illingworth [16] and proposed by different researchers for determining the condition index, it is here emphasized the methods which measure fresh and dry weight, volume and glycogen and solids percentage. We adopted in the present work the solidspercentage method formerly proposed by Shaw *et al *[17], with some modifications we had to do because of our laboratorial routine. Their method also allows dry weight measurements and consequently a broad comparison with results obtained from many different researchers. Solids percentage measurement which shows the molluscs condition index, is estimated from ratio 'meat dry weight: meat fresh weight' obtained from the bivalves. The results we obtained as compared to results obtained by Andrade and Leonel [18] and by Nishida *et al *[19] showed to be quite accurate, which is a reinforcement to the choice we made for this method.

### Data collection

From 2nd September to 10th October 1998 we carried out 20 catches or sampling of *unha-de-velho *during a full lunar-tide cycle. Molluscswere collected every two days by using a hoe and choosing only the intact specimens usually captured. Any chance of proceeding error would be minimized as we used two gatherers, one most skilled gatherer designated here as *pivô *and another gatherer who would substitute the *pivô *if needed. A subsample of 20 molluscs was selected at each sampling day, made up of individuals measuring from 5 cm to 6 cm length whichwere then subdivided into four sets with five animals each. Altogether, 400 animals were sampled and analyzed with respect to the parameters we proposed to study. Fresh and dry weight of each bivalve meat was individually determined at every set. Molluscus valves were open by cutting the abductormuscle with a blade. The shell cavity fluid was drained off and its excess was removed from the meat on an absorbent paper. The meat was then turned up on a new absorbent paper. A standard 10 minutes time was established for each set processed. Each bivalve meat was maintained in a previously weighted small box made with aluminium foil. After fresh weight determination the dry weight was determined by drying the meat in an oven at 60°C over 48 h, a sufficient period until the weight stabilization was obtained; a procedure that differs of the freeze-dry technique used by Shaw *et al *[17].

## Results and discussion

**Figure 2:**
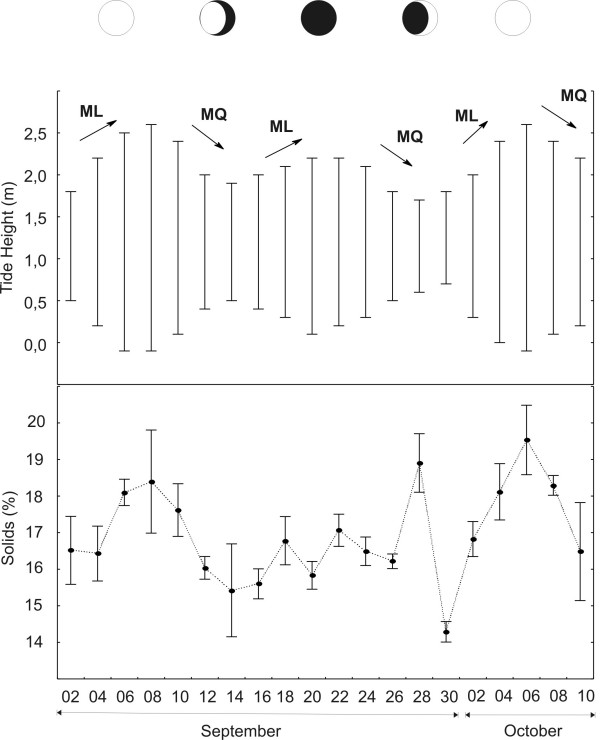
Condition index variation (solids percentage) of *unha-de-velho *or 'oldman's nail' (*Tagelus plebeius*) as a function of lunar-tide cycle. Abreviations: *ML – maré de lançamento *(or spring tide) and *MQ – maré de quebramento *(or neap tide). Twenty sampling of *unha-de-velho *during a full lunar-tide cycle. Each sample is composed by twenty individuals of *T. plebeius*.

In Fig. 2 the condition index curve of *unha-de-velho *is shown according to lunar phases and tide cycle, or simply lunar-tide cycle, and it is noteworthy the evidence of an overlapping of the empirical and the scientific approaches we observed. The gatherers' assertion *a unha tá gorda de acordo com a maré *(or the 'oldman's nail' is fat according to the tide) is corroborated from the condition index data obtained. As the high tides increase (spring tides) the condition index values also increase, and they decrease afterwards when the tide becomes lower (neap tide). Thegatherers' perception show to be quite accurate when we consider that 18 ofthe 20 catches we performed (80%) corresponded to the pattern described above. The remaining 20% (or just two catches) when this pattern was not confirmed, may be due, among other factors, to errors we made during our fieldwork or during the samples processing in the laboratory.

The molluscs distribution in the estuary is related to water salinity [20], water temperature [21] and to the amount of sediment particles in suspension [22], which are some of the abiotic factors responsible for the condition index variation. The latter seems to be particularly related to the gatherers' explanation. High spring tides show a strong dynamics and duringflood-tide the sea water frothes as it tumbles over a sandy-loam bank and the froth certainly contains flocculated planctonic microorganisms that feed the molluscs, thus increasing their weight.

Several authors studied the relation between molluscs condition index and their reproduction cycle and reported that after spawn the condition index decreases significantly [23-27]. This aspect was also confirmed by Bressan and Marin [28] and by Castro and Mattio [29] who related the condition index decrease to lipids and protein depletion. The gatherers who associate themolluscs condition (fat or thin) with the reproduction event also interpreted this phenomenon relating it to tidal fluctuations, as can be seen from their assertion *no quebramento para o lançamento o marisco engorda, fica completo dentro da caixinha e no quebramento o marisco emagrece ... no lançamento pro quebramento tem uma desova, quando desova é que ele tá magro *(roughly translated as: 'from neap tide to spring tide the mollusc becomes fat, it is full inside the valves and during neaptide it becomes thin ... from spring tide to neap tide there is a spawn, and then the mollusc is thin').

The size class pattern is necessary, as reported by Baird [30] because the condition index values may vary according to this parameter. At last, predation [31] and parasitism [32] are biotic interactions which may affectthe molluscs conditions. The latter mentioned authors with respect to parasitism, also studied *Tagelus plebeius *and reported that this mollusc was infected by a trematode belonging to family Gymnophallidae and related this fact to the bivalve condition index reduction whose lipid-protein reserve content was depleted by the parasite. We could observe during the condition index determination that some *unha-de-velho*specimens presented the internal parts of the valve with rusty colloured concretions, an indicator of parasitism. However, the infection level did not seem to be significant enough for affecting the bivalve condition index.

From our observations we conclude that several ecological parameters may, directly or indirectly, separately or jointly, affect the condition index. Although we recognize that further investigations are important for explaining and correlating the parameters here studied, we observed that the aim of the present work, relating an empirical observation to a scientific evaluation, was substantiated by the data we obtained.

Gatherers associate tidal variations to life cycle of different molluscs they exploit. They also recognize that the distribution of those animal species in mangrove habitats and estuaries is directly related to variationof tides. Gatherers' knowledge can provide a useful basis for understandinglocal mollusc stocks and their population dynamics.

Ethnoecological studies may also help in promoting dialogue and cooperationbetween fishers and scientists. The literature illustrates the significanceand value of traditional knowledge in Brazil. Alves and Nishida [33], for example, reported on the influence of tide variations on the ecdysis of themangrove crab *Ucides cordatus *in natural environments, based on information obtained from gatherers.
